# Mortality, Intensive Care Unit Admission, and Intubation among Hospitalized Patients with COVID-19: A One-Year Retrospective Study in Jordan

**DOI:** 10.3390/jcm12072651

**Published:** 2023-04-02

**Authors:** Khaled Al Oweidat, Rasmieh Al-Amer, Mohammad Y. Saleh, Asma S. Albtoosh, Ahmad A. Toubasi, Mona Khaled Ribie, Manar M. Hasuneh, Daniah L. Alfaqheri, Abdullah H. Alshurafa, Mohammad Ribie, Amira Mohammed Ali, Nathir Obeidat

**Affiliations:** 1 Department of Respiratory and Sleep Medicine, Faculty of Medicine, The University of Jordan, Amman 11942, Jordan; k.oweidat@ju.edu.jo (K.A.O.); nobeidat@ju.edu.jo (N.O.); 2 Department of Internal Medicine, Faculty of Medicine, The University of Jordan, Amman 11942, Jordan; asmaalbtoosh@gmail.com (A.S.A.); tubasi_ahmad@yahoo.com (A.A.T.); monakhald01@gmail.com (M.K.R.); manarmh25@gmail.com (M.M.H.); dania.laith1992@gmail.com (D.L.A.); shurafaabdullah@gmail.com (A.H.A.); mohamedribie@gmail.com (M.R.); 3 Faculty of Nursing, Isra University, Amman 11953, Jordan; 4 School of Nursing and Midwifery, Western Sydney University, Penrith, NSW 2751, Australia; 5 Faculty of Nursing, The University of Jordan, Amman 11942, Jordan; m.saleh@ju.edu.jo; 6 Department of Psychiatric Nursing and Mental Health, Faculty of Nursing, Alexandria University, Smouha, Alexandria 21527, Egypt; amira.mohali@alexu.edu.eg

**Keywords:** infectious diseases, COVID-19, intubation, intensive care, retrospective, Jordan

## Abstract

COVID-19 is a public health crisis that has caused numerous deaths, necessitated an increased number of hospital admissions, and led to extended inpatient stays. This study aimed to identify the factors associated with COVID-19 mortality, intensive care unit admission, intubation, and length of hospital stay among Jordanian patients. This was a one-year retrospective study of 745 COVID-19 patients admitted to Jordan University Hospital. Data regarding the patients’ demographics, clinical and co-morbid conditions, imaging, laboratory parameters, mortality, intensive care unit admission (ICU), and intubation were collected from their medical records using a coding manual. The data revealed that the overall rates of COVID-19-related mortality, ICU admission, and invasive intubation were 23.0%, 28.3%, and 10.8%, respectively. Chronic kidney disease (CKD), troponin, lactate dehydrogenase (LDH), and O_2_ saturation <90% were significantly associated with the mortality rate. The variables that were significantly associated with ICU admission were heart failure and the use of remdesivir. However, O_2_ saturation <90% and gastrointestinal (GI) symptoms were the only variables associated with invasive intubation. The findings of this study suggest that study-related health outcomes can be used to predict the severity of COVID-19, and they can inform future research aiming to identify specific populations who are at a higher risk of COVID-19 complications.

## 1. Introduction

The COVID-19 outbreak has been defined as a global pandemic caused by the severe acute respiratory syndrome coronavirus 2 (SARS-CoV-2) [1]. While there are indications that the pandemic could eventually become endemic, there have been resurgences of new virus variants over its course. These variants can affect the immunity acquired through vaccination or previous infection with COVID-19 [2].

SARS-CoV-2 is a novel RNA virus capable of inducing severe disease in multiple animal species, including humans. It belongs to the Coronavirus genus and the Coronaviridae family and shares structural traits with other viruses in this family, such as a single-stranded RNA genome encoding for spike, nucleocapsid, envelope, and membrane proteins [3,4]. Over time, the virus has undergone genetic changes, leading to the emergence of more pathogenic strains. Despite the high conservation of structural proteins among coronaviruses, with up to 90% similarity [3,4], even minor genetic variations can significantly impact the virus’s configuration and functionality. Consequently, a slight genetic alteration may cause a major shift in the arrangement of target proteins, rendering current treatments ineffective [3]. The virus spreads through direct and indirect contact, aerosols, and other means, with structural proteins on the virus’s surface playing vital roles in the development of complications.

COVID-19 can manifest with a broad range of clinical features that can be classified based on the severity of the disease [5]. According to the National Institutes of Health (USA), asymptomatic infection is defined as having no symptoms consistent with COVID-19. Mild illness can be classified as symptoms of COVID-19 without shortness of breath or abnormal chest imaging. Moderate illness includes evidence of lower respiratory dysfunction upon clinical assessment or imaging and oxygen saturation (SpO_2_) ≥94%. Severe illness is defined as SpO_2_ <94% in room air at sea level, a ratio of arterial partial pressure of oxygen to fraction of inspired oxygen (PaO_2_/FiO_2_) <300 mm Hg, a respiratory rate >30 breaths/min, or lung infiltrates >50%. Lastly, critical illness includes respiratory failure, septic shock, and/or multiple organ dysfunction [5].

The ability of SARS-CoV-2 to attach to the angiotensin-converting enzyme 2 (ACE2) receptor and affect multiple organs presents a significant risk of mortality to individuals with pre-existing comorbidities, including cardiovascular disease, diabetes, obesity, asthma, chronic obstructive pulmonary disorder, immune deficiencies, chronic renal impairment, and neurodegenerative diseases [6]. Such pre-existing conditions can worsen the severity and mortality of COVID-19 by compromising the metabolic and immune systems. As a result, there are strong correlations between these underlying medical conditions and COVID-19 [3,6].

In the early period of the pandemic, the mortality rate was high among hospitalized patients, approaching 32% [7]. Several studies have reported various predictors of the characteristics of COVID-19 patients, such as demographic, clinical, immunological, hematological, biochemical, and radiographic findings [8,9,10,11,12]. Demographic factors such as age and gender have been reported to impact COVID-19 outcomes. Older age has been associated with increased disease severity and a reduced likelihood of survival [8,9,10,11,12], and males were found to be more prone to severe clinical disease and increased mortality in comparison to their females counterparts [8]. Pre-existing comorbid conditions such as cardiovascular disease, diabetes, chronic respiratory disease, hypertension, and cancer have also been associated with increased case fatality rates [10,11,12,13,14,15,16]. In addition to high levels of C-reactive proteins (CRP), D-dimer, procalcitonin (PCT), total bilirubin, renal dysfunction indices, and interleukin-6 levels (IL-6), as well as low lymphocyte counts, were found to predict a severe disease course and death [13]. In terms of disease management, patients treated with methylprednisolone had lower mortality rates than those who were not treated with this drug [13].

In the Middle East, four studies evaluated predictors of COVID-19 severity and mortality [17,18,19,20]. One of these studies, conducted in Kuwait, showed that patients with heart disease, kidney disease, abnormal inflammatory markers, and abnormal coagulation had higher COVID-19 severity [17]. Another study conducted in Egypt showed that a history of smoking, ischemic heart diseases, and secondary bacterial pneumonia were predictors of COVID-19 severity [18]. Another study conducted in Oman showed that older adults and males had a higher risk of mortality [19]. Similarly, a study conducted in Saudi Arabia showed that aging and male gender, along with hypertension and diabetes, were predictors of mortality [20]. 

At the time of the writing of this report, specifically 27 January 2023, the number of confirmed cases in Jordan was 1,746,997, with 14,122 cumulative deaths. Meanwhile, the number of confirmed cases of COVID-19 in the Middle East was 23,239,808, globally, and the number of confirmed cases was 664,873,023, with 6,724,248 deaths [21].

Despite the high number of confirmed COVID-19 cases in Jordan, which rapidly increased during the first wave of the pandemic, indicating a difficult situation, data on the characteristics of patients with COVID-19—specifically, the predictors of mortality, ICU admission, and length of stay among patients—are scarce. In fact, only one study has been conducted in Jordan to assess the variables associated with the length of hospital stay among COVID-19 patients [22]. Although this study yielded essential data, it recruited a small sample size, which restricts the power and generalizability of the study findings. Moreover, this study was conducted at the beginning of the pandemic, when COVID-19 patients were admitted to hospitals regardless of the severity of their condition, which could have mitigated factors associated with the length of hospital stays. To our knowledge, no study conducted in Jordan has examined variables including the mortality, ICU admission, intubation, and length of hospital stay of Jordanian patients with COVID-19. Hence, we believe that our study closes this gap in the literature and provides rigorous data with robust power, as it reports on a large sample size examined within a one-year period [22]. 

Ethnicity and host genetics can impact COVID-19 susceptibility, outcomes [23], and, thus, the predictors of disease severity. Therefore, it is important to implement studies of a range of populations in order to best understand the predictors of the disease, thus aiding in the identification of patients at the highest risk of COVID-19 mortality and the construction of risk scores depending on these predictors. Although several studies have been conducted in the Middle East, it was demonstrated that the mortality impact of COVID-19 differs according to subregions [24]. Accordingly, the scarcity of COVID-19 patient outcome data in the Middle East necessitates further regional studies. It is worth noting that COVID-19 is a new disease, and our knowledge about it is still expanding and evolving. Hence, a study lasting one year may not be sufficient to provide comprehensive data related to the disease and its outcomes. It is also important to note that, in the case of an outbreak of a disease such COVID-19, one must track the disease and patients’ responses to it over time, which can be achieved by continuous monitoring and updating of the data. Hence, the main aim of this study was to identify risk factors associated with the mortality, ICU admission, intubation rate, and length of stay of patients hospitalized with COVID-19 in Jordan. 

## 2. Material and Methods

### 2.1. Design, Aim, and Setting

A one-year retrospective observational design was employed with consecutive sampling so as to include all COVID-19 patients who were admitted to Jordan University Hospital in Amman, Jordan, using the hospital’s electronic medical records (EMR). JUH is a tertiary hospital located in Amman, Jordan, which encompasses all major, sub-medical, and clinical specialties (amounting to 64 different specialties) and receives cases from all over Jordan. The main aim of this study was to identify the predictors of COVID-19-related mortality, intensive care unit admission, intubation, and length of hospital stay. 

### 2.2. Operational Definitions 

A Diagnosis of SARS-CoV-2 infection was confirmed by nasopharyngeal swab real-time reverse-transcriptase polymerase chain reaction (RT-PCR). Both the nucleic acid extraction kit and detection kit were supplied by Zybio, Inc., China). The RT-PCR samples were collected within the first two weeks of symptom onset.

Mortality of COVID-19-related death was defined as mortality that occurred within the first 28 days after admission and was attributed to COVID-19 complications.

The length of stay was defined as the time elapsed between a patient’s hospital admittance and discharge.

The presence of fever on admission was identified as an emergency room triage oral temperature higher than 37.7 °C [25], with hypoxia on admission determined by oxygen saturation and subsequently divided into three groups; <90%, 90–94%, and >94% in room air or ambient air.

Troponin values were considered positive if they were ≥45 pg/mL Hemoglobin A1c (HbA1C) within the last 3 months of admission was used to determine the degree of diabetic control among patients with diabetes, with an HbA1C ≥9 indicating uncontrolled diabetes [26].

Kidney injury was defined as an increase in serum creatinine to ≥1.5 times the baseline [27].

Smoking status was defined according to the WHO guidelines [28], according to which a current smoker is a person who smokes cigarettes daily or occasionally; a past smoker is a person who smoked in the past or smokes occasionally but has quit almost entirely; and a non-smoker is a person who has never smoked or who has smoked very rarely in the past.

Co-morbidities including, but not limited to, hypertension and diabetes were recorded according to the medical records as either “yes” or “no”.

### 2.3. Characteristics of the Study Population

Data were collected from 753 medical records of COVID-19 patients who met the following criteria: a confirmed diagnosis of SARS-CoV-2 infection and adult age ≥ 18 years. The exclusion criteria for this study were: (a) age less than 18 years and (b) pregnancy. All COVID-19 patients admitted to JUH between September 2020 and August 2021 who met the inclusion criteria were included in the study. 

### 2.4. Data Collection

The researchers developed a coding manual to collect the data. This method is the gold standard because it provides clear direction for the data collectors regarding how to collect each study-related variable using the data collection tool [29]. A team of physicians and medical students performed the data abstraction for this study, having previously received training on data abstraction. 

The coding manual included the following variables: demographic factors; signs and symptoms; co-morbid conditions; clinical, imaging, and laboratory parameters; mortality; treatments; intensive care unit admission; and intubation, which are briefly discussed in the following paragraph.

Demographic factors (age, sex, and smoking status) and the presentation of signs and symptoms (including a cough, shortness of breath, hypoxia, runny nose, fever, headache, and gastrointestinal symptoms) were assessed. Comorbidities were collected as present or not, including hypertension (HTN), diabetes mellitus (DM), cardiovascular disease (CVD), heart failure (HF), chronic kidney disease (CKD), malignancy, asthma, chronic obstructive pulmonary disease (COPD), dyslipidemia, neurological diseases, and autoimmune diseases. Radiological features were analyzed using the available inpatient records if present, including chest X-ray, high-resolution computed tomography (HRCT), and computed tomography pulmonary angiogram (CTPA).

Furthermore, laboratory data, including the patients’ venous blood sample laboratory results, were analyzed. The data included a complete blood count (CBC) (counts/deciliter), D-dimer (nanogram/milliliter), C-reactive protein (CRP) (milligram/liter), interleukin 6 (IL6) (picogram/milliliter), troponin I (picogram/milliliter), procalcitonin (nanogram/milliliter), absolute lymphocyte count, ferritin level (milligram/deciliter), serum creatinine (milligram/deciliter), urea (milligram/deciliter), and last hemoglobin A1c (HbA1C) within 3 months of admission. In addition, lactate dehydrogenase (LDH) (microgram/liter), aspartate aminotransferase (AST) (microgram/liter), alanine aminotransferase (ALT) (microgram/liter), serum potassium (milligram/deciliter), and serum sodium (milligram/deciliter) levels were also collected. Serum creatinine values were used to identify acute kidney injury (AKI) among the COVID-19 patients.

Treatment modalities, including medications received during admission (dexamethasone, antiviral therapy, tocilizumab) and oxygen delivery devices such as nasal cannula, face masks, venturi, non-rebreather masks, non-invasive ventilation, high-flow nasal cannula, and invasive mechanical ventilation, were also accounted for.

### 2.5. Ethical Statement

The study protocol was approved by the Institutional Review Board (IRB) of the University of Jordan (ethics number IRB#1020222444). Note that, in Jordan, waiving of consent is possible based on the approval of the ethics committee provided that the data are deidentified immediately after data collection and prior to analysis by the research team.

### 2.6. Statistical Analysis

This study included 753 medical records of COVID-19-positive patients admitted to our hospital, of which we excluded 8 records with missing data concerning health outcomes. Thus, the total sample for this study was 745. The data of the study participants were entered into Microsoft Office Excel 2019 spreadsheets and then imported into IBM SPSS v.25 software to conduct analyses. Counts and percentages were used to describe categorical variables. Means and standard deviations were used to describe normally distributed continuous variables, whereas medians and interquartile ranges were used to describe continuous variables that were not normally distributed. To identify the predictors of COVID-19-related mortality, ICU admission, and invasive intubation, binary logistic regression analysis was employed. In addition, univariable linear regression was conducted to identify the variables and predictors of the length of hospital stay. To adjust for confounding variables, significant variables associated with COVID-19-related mortality, ICU admission, and invasive intubation were incorporated into a multivariable binary logistic regression model. Variables significantly associated with the length of hospital stay were retested using multivariable linear regression so as to adjust for confounding variables. The results of the univariate binary and linear logistic regressions were expressed using the crude odds ratio (COR) and crude B coefficient (CB) with their corresponding 95% confidence intervals (95% CIs), respectively, while the results of the multivariate binary and linear logistic regression models were expressed using the adjusted odds ratio (AOR) and adjusted B coefficient (AB) with their corresponding 95% confidence intervals (95% CIs), respectively. All variables with a *p*-value <0.05 for the univariate and multivariate logistic regression models were considered statistically significant.

## 3. Results

### 3.1. Demographic and Clinical Characteristics and Co-Morbid Conditions of Jordanian Patients with COVID-19

As illustrated in Table 1, the sample of COVID-19 patients in our study was 745 patients (51.3%). The average age of the patients was 63.15, with S.D ± 15.99 years, and the majority of the patients were non-smokers (86.6%). Regarding COVID-19 severity, 28.3% of the patients were admitted to the ICU, and 10.8% of the patients were intubated. The most frequent complaints reported by the patients were shortness of breath (35.0%), cough (15.5%), and generalized weakness (15.0%). In addition, 30.4% of the patients reported gastrointestinal symptoms (226/744). Moreover, only 3.2% of the patients were vaccinated, and the majority (64.4%) of the patients were hospitalized in the second wave of the pandemic (Table 1). Concerning the outcomes of the COVID-19 patients, 23.0% of all the patients died (COVID-19-related death), while 53.1% of the patients who were admitted to the ICU died. The mean and standard deviation for the length of hospital stay were 10.09 ± 9.08 (Table 1). 

**Figure 1:**
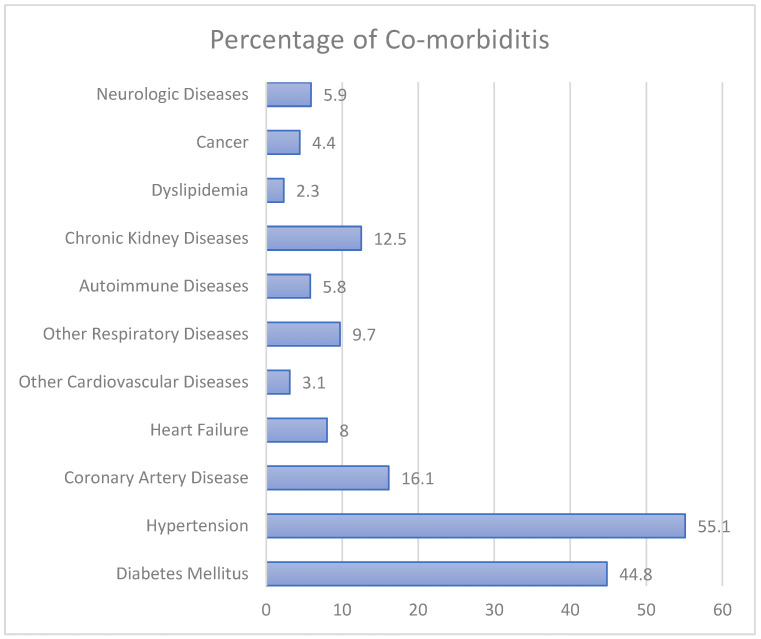
Comorbidities among COVID-19 patients (*n* = 745).

Regarding comorbidities, 44.8% of the patients had diabetes mellitus (DM) and 55.1% had hypertension (HTN). Furthermore, 16.1% of the patients had coronary artery disease (CAD) and 8% of the patients had heart failure (HF). Additionally, 12.5% and 5.9% of the patients had chronic kidney disease (CKD) and neurologic diseases, respectively (Figure 1).

### 3.2. Clinical, Laboratory, and Imaging Parameters of Jordanian COVID-19 Patients (n = 745)

We then analyzed the patients’ data to understand outcomes related to the clinical findings, imaging, and laboratory results. Only 22.5% of the patients had fever documented in hospital, although 47.2% reported fever prior to admission (see Table 1). The level of O_2_ saturation on admission was 90–94% in 24.4% of the patients and <90% in 41.9% (see Table 2). 

The most frequently used imaging methods were chest X-ray, high-resolution computed tomography (HRCT), and computed tomography pulmonary angiography (CTPA). The most frequently observed changes on chest X-ray were bilateral infiltrates (70.9%), whereas the most frequently observed changes on HRCT were ground glass opacifications (70.0%). 

Additionally, 14.0% had a positive CTPA indicating pulmonary embolism (PE). Regarding the laboratory investigations, troponin and D-dimer were positive in 21.1% and 85.7% of the patients, respectively. Additionally, the level of creatinine was higher than the cut-off point for the definition of acute kidney injury (AKI) in 30.5% of the patients, whereas the median and interquartile range of hemoglobin (Hb) and white blood cell (WBC) count were 13.05 [1.85] and “9.2 × 10^3^” [7.58 × 10^3^], respectively. Moreover, the median and interquartile range of ferritin and lactate dehydrogenase (LDH) were 330.95 [367.00] and 768 [406.00], respectively. The most frequently employed O_2_ delivery device was the nasal cannula, used for 49.7% of the patients. The second and third most frequently used O_2_ delivery devices were the simple face mask (27.3%) and non-rebreather mask (24.5%), respectively. Regarding the medications used for treating COVID-19 patients, systemic corticosteroids were used in 88.6% of the patients, with remdesivir used in 24.6% and tocilizumab used in 9.1%. Detailed data regarding the investigations and treatment methods of the patients are described in Table 3.

### 3.3. Predictors of COVID-19-Related Outcomes (Mortality, Intensive Care Unit Admission, and Intubation)

In the non-adjusted model, age, DM, HTN, CAD, HF, CKD, neurologic diseases, chest X-ray patterns, troponin, hemoglobin, WBCs, neutrophils, D-dimer, AKI, LDH, potassium, total bilirubin, and O_2_ saturation <90% were significantly associated with mortality. After adjustment for confounding variables, CKD (AOR = 3.831; 95% CI: 1.179–12.446), troponin (AOR = 3.060; 95% CI: 1.156–8.102), LDH (AOR = 1.002; 95% CI: 1.001–1.003), and O_2_ saturation <90% (AOR = 2.761; 95% CI: 1.066–7.155) were the only variables significantly associated with mortality (Table 4). Patients who had CKD, positive troponin, high LDH values, and O_2_ saturation <90% had a significantly higher risk of mortality. 

In the non-adjusted model that was used to investigate ICU admission, factors including age, smoking status, GI symptoms, DM, HTN, CKD, HF, other CVDs, chest X-ray patterns, troponin, GI symptoms, neutrophils, D-dimer, AKI, potassium, sodium, O_2_ saturation <90%, tocilizumab use, and remdesivir use were significantly associated with ICU admission. However, in the adjusted model, only HF (AOR = 7.894; 95% CI: 1.391–44.818) and remdesivir use (AOR = 0.192; 95% CI: 0.044–0.835) were significantly associated with ICU admission (Table 5). 

Patients who had HF had a significantly higher risk of ICU admission, while patients who were treated with remdesivir had a significantly lower risk of ICU admission. Furthermore, in the non-adjusted model that was used to evaluate invasive intubation, factors including GI symptoms, CKD, neurological diseases, chest X-ray patterns, troponin, D-dimer, AKI, sodium, ALT, AST, O_2_ saturation <90%, and steroids were significantly associated with invasive intubation. In the adjusted model, O_2_ saturation <90% (AOR = 16.585; 95% CI: 2.892–95.118) and GI symptoms (AOR = 0.323; 95% CI: 0.105–0.993) were the only variables significantly associated with invasive intubation (Table 6). Patients who had O_2_ saturation <90% had a significantly higher risk of invasive intubation, whereas patients who reported GI symptoms had a significantly lower risk. 

Turning to the length of hospital stay, the mean and standard deviation were 10.09 ± 9.08 (Table 1). In the non-adjusted model, pregnancy, chest X-ray patterns, Hb, D-dimer, total bilirubin, O_2_ saturation, Tocilizumab use, and remdesivir use were significantly associated with the length of hospital stay. However, after adjusting for confounding variables, none of the aforementioned variables were significantly associated with the length of hospital stay (Table 7).

## 4. Discussion

The main aim of this study was to identify the predictors of the mortality rate, ICU admission, and intubation rate among Jordanian patients with COVID-19. Additionally, this study reported on the demographic and clinical characteristics, co-morbid conditions, and imaging and laboratory parameters of patients with COVID-19. The sample in this study were all Jordanian adults who were admitted to JUH with COVID-19. 

Our study showed that the most frequent symptoms of COVID-19 among the patients were shortness of breath (35.0%), cough (15.5%), and generalized fever (15.0%). The reported prevalence of COVID-19 symptoms varies between studies. Studies from Brazil [30], Saudi Arabia [31], Kuwait [17], and China [32] reported a higher prevalence of fever and cough, with fever being the most common symptom among their patients. 

In addition, we reported a low percentage of asymptomatic patients (<1%), whereas studies conducted in the USA [33], Kuwait [17], and China [34] reported that approximately 40% of their patients were asymptomatic. However, these differences can be explained by the fact that the majority of these studies were conducted at the beginning of the pandemic, when most COVID-19 patients were admitted to the hospital regardless of their symptom severity. A potential explanation for this trend is that the virus can mutate, leading to different variants that may have different symptoms. Variants of the virus have been identified in different regions of the world, and some variants may be more transmissible or more likely to cause severe illness than others. Additionally, the population’s susceptibility to the disease may vary by region. People living in different regions may have different genetic or environmental factors that affect their risk of developing severe illness from COVID-19. Additionally, the means by which the disease is diagnosed and reported can also vary by region and lead to differences in the reported symptoms. Lastly, the level of access to healthcare and the means by which healthcare is delivered can also affect the symptoms reported. In regions where healthcare systems are not well-developed, people may not have access to the same level of care, and symptoms may be reported differently compared to regions with well-developed healthcare systems.

Furthermore, our findings demonstrate that the most common radiological findings on the chest X-rays of our COVID-19 patients were bilateral infiltrates (70.9%). Similarly, studies conducted in China [32] and the United Kingdom [35] also reported that the most common finding on chest X-rays of COVID-19 patients was bilateral infiltration. In addition, most of the patients in our study who underwent HRCT imaging had ground glass opacification (70.0%), which is consistent with previous studies conducted in China [32]. More than 50% of the patients in our cohort required non-invasive oxygen support systems, with face masks and non-rebreather masks being the most commonly used devices, while only 10.8% of the patients in our study required invasive intubation. The rates of non-invasive oxygen support system use were similar to those reported in previous studies [35].

However, the rates of intubation in our cohort were lower compared to those in previous studies, which reported that approximately 20% of admitted patients required invasive intubation [8]. This low rate of intubation might be a consequence of the refusal of intubation by the patients, which could possibly be related to cultural or religious beliefs; for example, patients may have strong beliefs against certain medical procedures or may view intubation as a last resort. Another reason could be fear of the procedure and its potential complications, such as infection, injury to the vocal cords, or difficulties with weaning from the ventilator. Additionally, the fear of being in the hospital for a long period of time or not recovering could also be a reason for refusing intubation. It is also worth noting that, in some cases, patients may not fully understand the risks and benefits of intubation and may make decisions based on incomplete or inaccurate information. It is important for healthcare providers to engage in open and transparent communication with patients and their families and to provide them with accurate information about the risks and benefits of intubation and other treatment options. This can help the patients and their families to make informed decisions about their care. It is also important to respect patients’ autonomy and to provide them with the best possible care in line with their own values and beliefs.

Moreover, the mortality rate in our cohort was 23%, which is quite similar to the rates reported in previous studies conducted in Kuwait [17], Spain [36], and Germany [37]. The ICU mortality rate in our study (53.1%) is consistent with those reported in several studies conducted in Italy [38], Germany [37], Saudi Arabia [31], and Kuwait [17]. However, our study identified high rates of comorbidities, as almost half of our study cohort had diabetes and hypertension. On the other hand, in the studies conducted in Kuwait [17] and Saudi Arabia [31], only around 25% and 10% of the patients had diabetes and hypertension, respectively.

Regarding the variables associated with COVID-19-related mortality and severity, CKD, positive troponin, LDH, and O_2_ saturation below 90% were significantly associated with a higher risk of COVID-19 mortality. Previous studies showed that positive troponin was a significant predictor of COVID-19 mortality, indicating that cardiovascular injury is associated with higher mortality among COVID-19 patients [39]. However, in contrast to our study, others showed that another cardiac marker, BNP, was also associated with a higher risk of mortality [40]. Additionally, LDH was significantly associated with a higher risk of mortality, a finding that is consistent with previous studies showing that higher LDH levels were independent predictors of mortality [39]. LDH is an enzyme that is found in many organs, with its elevation indicating multiorgan injury [41]. Diabetes and hypertension were not identified as predictors of mortality, and contradicting the results of several studies [17], our study showed that CKD, which is a common complication of hypertension and diabetes, was significantly associated with COVID-19 mortality. A comprehensive study of the implications of potential biomarkers reported that adiponectin plays essential roles in controlling glucose metabolism, insulin sensitivity, and fatty acid oxidation. Its role in viral infections is linked to its ability to regulate the immune response through its anti-inflammatory or pro-inflammatory axis [42]. Patients with CKD often have multiple comorbidities, such as hypertension and diabetes, which are also risk factors for severe COVID-19.

Similar to our study, several articles showed that low O_2_ saturation at presentation is associated with higher COVID-19 mortality, as low O_2_ saturation is associated with a higher degree of lung injury and delayed presentation [43]. Additionally, our analysis showed that low O_2_ saturation was significantly associated with invasive intubation. Surprisingly, the results revealed that presentation with GI symptoms was associated with a lower risk of invasive intubation. A meta-analysis showed that the prevalence of GI symptoms among COVID-19 patients was 20%, and it was not associated with a higher risk of mortality [44]. In addition, a cohort study showed that patients who presented with GI symptoms had significantly lower COVID-19 severity [45]. Our analysis revealed that heart failure and the use of remdesivir for the treatment of patients were the only significant factors associated with ICU admission. Other studies showed that one in four patients with heart failure who were hospitalized with COVID-19 infection died [46]. Moreover, consistent with our findings, cohort studies showed that the use of remdesivir reduced the rate of COVID-19 ICU admission but not mortality [47,48]. Confirmation of the clinical efficacy and safety profiles of favipiravir (FVP) and remdesivir (RDV) has established them as potential therapeutic candidates for the treatment of severe acute respiratory syndrome coronavirus 2 (SARS-CoV-2) infection [3].

It is important to mention that, in our study, age and sex were not significantly associated with higher COVID-19 severity or mortality. The majority of the studies in the literature showed that older age is a significant predictor of COVID-19 severity and mortality [49]. However, our findings can be explained by the fact that half of our patients were above 65 years old, with a mean age of 63.15 for the whole cohort, which limits the potential for comparison between the COVID-19 outcomes of elderly and young patients. On the other hand, the results concerning the association between COVID-19 mortality and sex in the literature are inconsistent, with some studies reporting a higher risk of mortality among the male sex [50] and others reporting a lack of significant differences in the rate of COVID-19 mortality between males and females [51]. Despite the fact that several studies showed that the use of steroids (dexamethasone and methylprednisolone) among COVID-19 patients reduced COVID-19 severity and mortality [52], our study showed no such association. However, in our study, steroids were used to treat a very high percentage of patients (88.6%), which limits the potential for comparison between the outcomes of patients who were treated with steroids and those who were not.

Despite the contributions of this study, it still had some limitations that need to be addressed in future research. Firstly, the study was carried out at a single center in Jordan, which may restrict the applicability of the results to other healthcare settings. However, we believe that the findings can still be reasonably generalized, because the study was conducted in a tertiary center that treats patients from all over Jordan, using a large database. Secondly, although our analysis was adjusted for confounding variables, the risk of confounding bias cannot be totally excluded. Third, while other studies have been conducted on this topic, this study is unique in that it was conducted in a different geographical location, namely, Jordan. This study thus provides valuable insights that complement the existing literature and contribute to a more comprehensive understanding of the subject from a different geographical perspective. To overcome the limitations mentioned above, future research could explore additional healthcare settings and use prospective designs to collect data on COVID-19 outcomes. Future studies could also investigate the impacts of comorbidities on outcomes of interest and identify potential risk factors that may contribute to poor outcomes among COVID-19 patients. Although this study provides valuable contributions to the existing literature on COVID-19 outcomes, further investigations are necessary so as to advance our understanding of the disease and its impacts on patients.

## 5. Conclusions

To conclude, we provided detailed information on the clinical characteristics, investigations, and treatment methods of 745 COVID-19 patients. The overall rates of COVID-19-related mortality, ICU admission, and invasive intubation were 23.0%, 28.3%, and 10.8%, respectively. Our study found that CKD, positive troponin, LDH, and O_2_ saturation <90% upon admission were significantly associated with COVID-19 mortality. Moreover, heart failure patients had a significantly higher risk of ICU admission, while remdesivir use for the treatment of COVID-19 patients was associated with a reduction in the risk of ICU admission. Patients who had O_2_ saturation <90% upon admission had a significantly higher risk of invasive intubation, whereas patients presenting with GI symptoms had a significantly lower risk.

## Figures and Tables

**Table 1:** Demographics of admitted COVID-19 patients (*n* = 745).

Variable	
Age, mean (*SD*), years	63.15 ± 15.99
Sex	
Male, *n* (%)	382 (51.3)
Female, *n* (%)	363 (48.7)
Smoking status	
Non-smoker, *n* (%)	645 (86.6)
Smoker, *n* (%)	60 (8.1)
Ex-smoker, *n* (%)	40 (5.4)
Admission	
Floor, *n* (%)	524 (71.7)
Intensive care unit, *n* (%)	207 (28.3)
Discharge, *n* (%)	547 (74.7)
Vaccination status	
Not vaccinated, *n* (%)	721 (96.8)
First shot, *n* (%)	265 (35.6)
Second shot, *n* (%)	480 (64.4)
Chief Complaints	
Generalized weakness, *n* (%)	110 (15.0)
Shortness of breath, *n* (%)	256 (35.0)
Cough, *n* (%)	113 (15.5)
Fever, *n* (%)	89 (12.2)
GI symptoms, *n* (%)	50 (6.8)
Chest pain	27 (3.7)
Asymptomatic, *n* (%)	4 (0.5)
Others, *n* (%)	71 (9.7)
Fatigue	
Yes, *n* (%)	366 (49.6)
Fever reported/prior to admission	
Yes, *n* (%)	348 (47.2)
Chills	
Yes, *n* (%)	265 (35.9)
GI Symptoms	
Yes, *n* (%)	226 (30.4)
Headache	
Yes, *n* (%)	82 (11.0)
Nasal Discharge	
Yes, *n* (%)	34 (4.6)
Sore throat	
Yes, *n* (%)	60 (8.1)
Chest pain	
Yes, *n* (%)	207(27.8)
Shortness of Breath	
Yes, *n* (%)	455(61.2)
Cough	
Yes, *n* (%)	492(66.6)
COVID-19-related health outcomes	
COVID-19-related death, *n* (%)	171 (23.0)
Non-COVID-19-related death, *n* (%)	14 (2.3)
Discharge, *n* (%)	97 (46.9)
Outcomes among ICU patients	
COVID-19-related death, *n* (%)	110 (53.1)

*n*: number; %: percentage; M: Mean; SD: Standard deviation.

**Table 2:** Clinical Findings, Laboratory Investigations, and Treatments (*n* = 745).

Variable	
**Chest X-ray**	
Clear, *n* (%)	82 (11.7)
Chest X-ray, *n* (%)	122 (17.4)
Bilateral infiltrate, *n* (%)	496 (70.9)
**High-Resolution Computed Tomography**	
Clear, *n* (%)	3 (5.0)
Ground glass, *n* (%)	70 (70.0)
Fibrotic changes, *n* (%)	25 (25.0)
**Computed Tomography Pulmonary Angiography**	
Negative, *n* (%)	37 (86.0)
Positive, *n* (%)	6 (14.0)
**Troponin**	
Negative, *n* (%)	377 (78.9)
Positive, *n* (%)	101 (21.1)
**D-Dimer**	
Negative, *n* (%)	91 (14.3)
Positive, *n* (%)	546 (85.7)
**Acute Kidney Injury (Creatinine)**	
Negative, *n* (%)	511 (69.5)
Positive, *n* (%)	224 (30.5)
**Last HBA1C**	
Controlled, *n* (%)	190 (87.6)
Uncontrolled, *n* (%)	27 (12.4)
**O_2_ Saturation**	
≥94, *n* (%)	239 (33.7)
90–94, *n* (%)	173 (24.4)
<90, *n* (%)	297 (419)
**Documented Fever**	
Yes, *n* (%)	578 (77.5)
No, *n* (%)	168 (22.5)
**Treatments Used**	
Steroids, *n* (%)	656 (88.6)
Tocilizumab, *n* (%)	68 (9.1)
Remdesivir, *n* (%)	183 (24.6)

**Table 3:** Laboratory Investigations and Treatments presented as median and interquartile range (*n* = 745).

Variables	
IL-6 (pg/mL), median (IQR)	13.00 (22)
Brain natriuretic peptide (pg/mL)	74.75 (75)
Pro-calcitonin (ng/mL)	0.10 (0)
Hemoglobin (g/dL)	13.05 (1.85)
White blood cell count	9.2 × 10^3^ (7.58 × 10^3^)
Neutrophil absolute count	7.97× 10^3^ (6.63 × 10^3^)
Lymphocyte absolute count	0.85 × 10^3^ (0.42 × 10^3^)
Platelet count	189.00 × 10^3^ (190.00 × 10^3^)
C-Reactive protein (mg/L)	99.95 (138.35)
Ferritin (ng/mL)	330.95 (367.00)
Lactate dehydrogenase (u/L)	768.00 (406.00)
Potassium (mg/dL)	4.30 (0.98)
Sodium (mg/dL)	139.50 (8.80)
Urea (mg/dL)	61.55 (40.50)
Alanine transaminase (u/L)	31.00 (22.00)
Aspartate transaminase (u/L)	40.00(53.00)
Total bilirubin mg/dL	0.50 (0.00)
Direct bilirubin mg/dL	0.20 (0.00)

**Table 4:** Regression Analysis of Predictors of COVID-19-Related Mortality (*n* = 745).

		COVID-19-Related Mortality			
Variable	Response	Correlation Coefficient	(95% CI)	Adjusted Odds Ratio (OR)	(95% CI)
Age		1.047	(1.033–1.061) *	1.013	(0.984–1.042)
Gender	Male	1.067	(0.764–1.489)		
	Female				
Smoking status	Smoker	0.523	(0.252–1.088)		
	Ex-smoker	1.566	(0.798–3.071)		
Chief complaints	Fatigue	R	R	R	R
	SOB	0.772	(0.471–1.265)	0.572	(0.201–1.633)
	Cough	0.544	(0.293–1.010)	0.433	(0.112–1.671)
	Fever	0.372	(0.182–0.761)	0.824	(0.231–2.937)
	Atypical	1.183	(0.627–2.230)	1.013	(0.220–4.670)
	GI symptoms	0.558	(0.250–1.248)	0.484	(0.111–2.120)
	Chest pain	0.622	(0.230–1.680)	0.928	(0.163–5.280)
	Asymptomatic	-	-	-	-
	Headache	0.544	(0.059–5.053)		
	Runny nose				
GI symptoms	Yes	0.722	(0.495–1.052)		
Diabetes mellitus	-	1.497	(1.071–2.092)	1.220	(0.567–2.623)
Hypertension	-	1.738	(1.229–2.457)	1.123	(0.484–2.606)
Coronary artery disease	-	1.582	(1.033–2.423)	0.734	(0.308–1.752) *
Chronic kidney disease	-	3.501	(2.229–5.499)	3.831	(1.179–12.446)
Asthma	-	0.851	(0.397–1.823)	-	-
COPD	-	0.434	(0.128–1.479)	-	-
Dyslipidemia	-	0.628	(0.178–2.209)	-	-
Cancer	-	0.985	(0.435–2.232)	-	-
Neurologic diseases	-	3.056	(1.639–5.698) *	0.843	(0.197–3.617)
Heart failure	-	4.191	(2.423–7.249)	0.956	(0.298–3.065)
Autoimmune diseases	-	1.462	(0.755–2.832)	-	-
Other respiratory disease	-	1.462	(0.755–2.832)	-	-
Other cardiovascular diseases	-	1.281	(0.576–2.849)		
Documented fever		1.132	(0.765–1.675)		
Chest X-ray					
	Unilateral changes	3.422	(1.621–7.360)	3.995	(0.744–21.456)
	Bilateral changes	3.454	(1.621–7.360) *	2.187	(0.488–9.797)
Computerized tomographic pulmonary angiography	Positive	0.540	(0.056–5.208)		
IL-6	-	1.004	(0.993–1.014)		
BNP	-	1.001	(1.000–1.001)		
Troponin	-	4.641	(2.917–7.383)	3.060	(1.156–8.102) *
Procalcitonin	-	0.788	(0.728–0.852) *	0.896	(0.759–1.058)
Hemoglobin	-	0.788	(0.728–0.852) *	0.896	(0.759–1.058)
White blood cells	-	1.035	(1.009–1.062) *	0.835	(0.613–1.138)
Neutrophils	-	1.125	(1.084–1.167) *	1.306	(0.933–1.828)
Lymphocytes		1.013	(0.971–1.058)		
Platelets		1.000	(1.000–1.001)		
CRP		1.000	(1.000–1.000)		
Ferritin		1.001	(1.000–1.001)		
D-dimer		3.680	(1.803–7.509) *	0.945	(0.270–3.305)
LDH		1.001	(1.001–1.002) *	1.002	(1.001–1.003) *
Creatinine		3.756	(2.640–5.343) *	1.128	(0.448–2.840)
Potassium		1.008	(1.002–1.014) *	0.986	(0.922–1.056)
Sodium		1.001	(0.995–1.006)		
Urea		1.000	(1.000–1.000)		
ALT		1.000	(0.999–1.002)		
AST		1.002	(1.000–1.003)		
Bilirubin		1.222	(1.058–1.410) *	0.837	(0.450–1.555)
Direct bilirubin		1.001	(0.995–1.008)		
Last HBA1C		2.146	(0.886–5.197)		
O_2_ saturation					
	≥94	R	R	R	
	90–94	1.186	(0.708–1.986)	1.558	(0.515–4.711)
	<90	2.630	(1.729–3.998) *	2.761	(1.066–7.155) *
Steroids	Yes	1.609	(0.896–2.890)		
Tocilizumab	Yes	1.584	(0.930–2.698)		
Remedisvir	Yes	0.916	(0.621–1.352)		

* *p*-value < 0.05, R: Reference group, COR: Crude odds ratio, AOR: Adjusted odds ratio, CB: Crude B coefficient, AB: Adjusted B coefficient.

**Table 5:** Regression Analysis of Predictors of ICU Admission due to COVID-19 (*n* = 745).

		COVID Related ICU Admission			
Variable	Response	Correlation Coefficient	(95% CI)	Adjusted Odds Ratio (OR)	(95% CI)
Age		1.034	(1.021–1.046) *	0.983	(0.936–1.033)
Gender	Male	0.968	(0.702–1.336)		
	Female				
Smoking status	Smoker	0.643	(0.333–1.238)	0.796	(0.088–7.169)
	Ex-smoker	2.203	(1.147–4.234) *	1.174	(0.077–17.827)
Chief complaints	Fatigue	R	R	R	R
	SOB	1.115	(0.680–1.828)		
	Cough	0.762	(0.416–1.393)		
	Fever	0.888	(0.473–1.669)		
	Atypical	1.216	(0.625–2.366)		
	GI symptoms	0.920	(0.431–1.964)		
	Chest pain	0.765	(0.281–2.083)		
	Asymptomatic	-	-		
	Headache	1.699	(0.271–10.663)		
	Runny nose	-	-		
GI symptoms	Yes	0.584	(0.402–0.848)	0.316	(0.056–1.791)
Diabetes mellitus	-	1.967	(1.420–2.724) *	1.136	(0.299–4.310)
Hypertension	-	1.675	(1.201–2.336) *	0.638	(0.126–3.227)
Coronary artery disease	-	1.066	(0.692–1.642)		
Chronic kidney disease	-	2.542	(1.627–3.972) *	0.637	(0.077–5.306)
Asthma	-	0.748	(0.349–1.605)		
COPD	-	0.890	(0.346–2.290)		
Dyslipidemia	-	1.799	(0.675–4.791)		
Cancer	-	0.670	(0.286–1.569)		
Neurologic diseases	-	1.714	(0.909–3.230)		
Heart failure	-	4.239	(2.452–7.330) *	7.894	(1.391–44.818)
Autoimmune diseases	-	0.755	(0.365–1.562)		
Other respiratory disease	-	1.127	(0.664–1.914)		
Other cardiovascular diseases	-	3.052	(1.461–6.372) *	0.340	(0.018–6.556)
Documented fever		1.096	(0.750–1.600)		
Chest X-ray					
	Unilateral changes	2.884	(1.295–6.423) *	2.245	(0.165–30.617)
	Bilateral changes	3.554	(1.730–7.300) *	1.465	(0.126–16.988)
Computerized tomographic pulmonary angiography	Positive	1.350	(0.213–8.551)		
IL-6	-	1.000	(0.989–1.010)		
BNP	-	1.000	(1.000–1.001)		
Troponin	-	3.384	(2.139–5.352) *	0.655	(0.118–3.620)
Procalcitonin	-	1.013	(0.971–1.058)		
Hemoglobin	-	0.853	(0.792–0.918) *	0.771	(0.554–1.072)
White blood cells	-	1.013	(0.996–1.030)		
Neutrophils	-	1.058	(1.025–1.092) *	1.003	(0.984–1.023)
Lymphocytes		1.011	(0.969–1.055)		
Platelets		1.000	(1.000–1.001)		
CRP		1.000	(0.999–1.000)		
Ferritin		1.001	(1.000–1.001)		
D-dimer		2.274	(1.268–4.077) *	1.907	(0.087–41.862)
LDH		1.001	(1.000–1.001)		
Creatinine		2.327	(1.657–3.268) *	0.901	(0.176–4.608)
Potassium		1.013	(1.006–1.019) *	1.051	(0.951–1.161)
Sodium		0.992	(0.987–0.997) *	1.024	(0.932–1.125)
Urea		1.001	(1.000–1.000)		
ALT		1.001	(0.999–1.003)		
AST		1.000	(0.999–1.001)		
Bilirubin		1.093	(0.959–1.245)		
Direct bilirubin		1.006	(1.000–1.013)		
Last HBA1C		0.987	(0.349–2.788)		
O_2_ saturation					
	≥94	R	R	R	R
	90–94	1.023	(0.616–1.698)	1.020	(0.141–7.397)
	<90	2.945	(1.965–4.412) *	0.938	(0.162–5.417)
Steroids	Yes	0.782	(0.471–1.300)		
Tocilizumab	Yes	2.479	(1.494–4.114) *	1.675	(0.294–9.524)
Remedisvir	Yes	0.591	(0.414–0.845) *	0.192	(0.044–0.835) *

* *p*-value < 0.05, R: Reference group, COR: Crude odds ratio, AOR: Adjusted odds ratio, CB: Crude B coefficient, AB: Adjusted B coefficient.

**Table 6:** Regression Analysis of Predictors of Intubation (*n* = 745).

		COVID-19-Related Mortality			
Variable	Response	Correlation Coefficient	(95% CI)	Adjusted Odds Ratio (OR)	(95% CI)
Age		1.012	(0.997–1.028)		
Gender	Male	1.121	(0.704–1.785)		
	Female				
Smoking status	Smoker	1.168	(0.510–2.676)		
	Ex-smoker	2.212	(0.978–5.002)		
Chief complaints	Fatigue	R	R		
	SOB	1.143	(0.578–2.261)		
	Cough	0.658	(0.269–1.610)		
	Fever	0.539	(0.196–1.482)		
	Atypical	1.368	(0.576–3.249)		
	GI symptoms	0.318	(0.069–1.465)		
	Chest pain	0.597	(0.126–2.819)		
	Asymptomatic				
	Headache	1.865	(0.193–17.992)		
	Runny nose				
GI symptoms	Yes	0.538	(0.304–0.954) *	0.323	(0.105–0.993) *
Diabetes mellitus	-	1.177	(0.740–1.873)		
Hypertension	-	1.093	(0.684–1.748)		
Coronary artery disease	-	0.634	(0.308–1.306)		
Chronic kidney disease	-	2.074	(1.153–3.728) *	3.640	(0.895–14.802)
Asthma	-	0.656	(0.198–2.178)		
COPD	-	1.776	(0.589–5.356)		
Dyslipidemia	-				
Cancer	-	1.188	(0.406–3.478)		
Neurologic diseases	-	4.075	(2.028–8.188) *	0.733	(0.144–3.732)
Heart failure	-	0.754	(0.344–1.651)		
Autoimmune diseases	-	0.604	(0.182–1.999)		
Other respiratory disease	-	1.035	(0.477–2.245)		
Other cardiovascular diseases	-	1.283	(0.436–3.776)		
Documented fever		1.163	(0.679–1.993)		
Chest X-ray					
	Unilateral changes	4.558	(1.297–16.016) *	6.307	(0.766–51.925)
	Bilateral changes	3.375	(1.031–11.048) *	1.820	(0.259–12.790)
Computerized tomographic pulmonary angiography	Positive				
IL-6	-	1.006	(0.995–1.017)		
BNP	-	1.000	(1.000–1.001)		
Troponin	-	2.974	(1.693–5.223) *	1.544	(0.563–4.234)
Procalcitonin	-	1.034	(0.989–1.081)		
Hemoglobin	-	0.812	(0.733–0.899)	0.917	(0.752–1.118)
White blood cells	-	1.001	(0.997–1.005)		
Neutrophils	-	1.002	(0.997–1.006)		
Lymphocytes		0.931	(0.769–1.128)		
Platelets		0.999	(0.997–1.000)		
CRP		1.000	(1.000–1.000)		
Ferritin		1.001	(1.000–1.001)		
D-dimer		2.951	(1.048–8.310) *	2.766	(0.310–24.703)
LDH		1.001	(1.001–1.002) *	1.001	(1.000–1.002)
Creatinine		2.306	(1.440–3.693) *	1.856	(0.643–5.357)
Potassium		0.926	(0.843–1.017)		
Sodium		1.027	(1.007–1.047) *	1.046	(0.992–1.103)
Urea		1.000	(1.000–1.000)		
ALT		1.004	(1.002–1.007) *	1.005	(0.996–1.014)
AST		1.003	(1.001–1.005) *	1.001	(0.999–1.003)
Bilirubin		1.090	(0.939–1.264)		
Direct bilirubin		0.954	(0.900–1.011)		
Last HBA1C		0.764	(0.167–3.495)		
O_2_ saturation		-	-	-	-
	≥94	R	R	R	R
	90–94	1.666	(0.750–3.697)	5.536	(0.771–39.750)
	<90	4.015	(2.089–7.715) *	16.585	(2.892–95.118) *
Steroids	Yes	5.533	(1.334–22.947) *	0.551	(0.077–3.940)
Tocilizumab	Yes	1.483	(0.725–3.032)		
Remedisvir	Yes	1.094	(0.645–1.856)		

* *p*-value < 0.05, R: Reference group, COR: Crude odds ratio, AOR: Adjusted odds ratio, CB: Crude B coefficient, AB: adjusted B coefficient.

**Table 7:** Regression Analysis of Predictors of the Length of Hospital Stay (*n* = 745).

		COVID-19 Related Length of Hospital Stay			
Variable	Response	(CB (95% CI)	(95% CI)	AB (95% CI)	(95% CI)
Age		0.015	(−0.027–0.057)		
Gender	Male	−1.158	(−2.478–0.162)		
	Female				
Smoking status	Smoker	0.945	0.945		
	Ex-smoker	0.730	(−0.924–1.109)		
Chief complaints	Fatigue	−0.500	(−1.077–0.177)		
	SOB	1.062	(2.358–−0.299)		
	Cough	−0.652	(−1.370–0.120)		
	Fever	0.334	(−0.344–1.012)		
	Atypical	0.211	(−0.930–1.945)		
	GI symptoms	−1.260	(−1.992–0.023)		
	Chest pain	0.444	(−1.203–1.975)		
	Asymptomatic	−0.399	(−1.485–0.310		
	Headache	−1.205	(−2.354–0.132)		
	Runny nose	−1.762	(−2.214–0.256)		
GI symptoms	Yes	−1.260	(−2.697–0.178)		
Diabetes mellitus	-	0.766	(−0.562–2.094)		
Hypertension	-	0.994	(−0.335–2.323)		
Coronary artery disease	-	0.781	(−1.006–2.568)		
Chronic kidney disease	-	−0.395	(−2.384–1.595)		
Asthma	-	0.011	(−2.961–2.984)		
COPD	-	−2.163	(−5.940–1.613)		
Dyslipidemia	-	0.455	(−3.933–4.823)		
Cancer	-	−1.052	(−4.229–2.126)		
Neurologic diseases	-	2.074	(−0.726–4.875)		
Heart failure	-	1.372	(−1.048–3.793)		
Autoimmune diseases	-	1.491	(−1.342–4.325)		
Other respiratory disease	-	−0.681	(−3.877–2.775)		
Other cardiovascular diseases	-	−0.551	(−3.877–2.775)		
Documented fever		0.724	(−0.848–2.296)		
Chest X-ray					
	Unilateral changes	0.856	(−0.226–1.976)		
	Bilateral changes	1.129	(0.140–2.118) *	0.631	(−1.075–2.337)
Computerized tomographic pulmonary angiography	Positive	−10.207	(−21.663–1.248)		
IL-6	-	−0.023	(−0.063–0.016)		
BNP	-				
Troponin	-	−0.607	(−2.708–1.494)		
Procalcitonin	-	0.198	(−0.004–0.400)		
Hemoglobin	-	−0.318	(−0.618–−0.019)	0.164	(−0.323–0.651)
White blood cells	-	0.002	(−0.014–0.019)		
Neutrophils	-	0.004	(−0.014–0.023)		
Lymphocytes		−0.051	(−0.241–0.139)		
Platelets		0.003	(0.000–0.005)		
CRP		0.001	(0.000–0.002)		
Ferritin		0.002	(0.001–0.004)		
D-dimer		2.398	(0.543–4.254) *		
LDH		9.588 × 10^−5^	(−0.001–0.002)		
Creatinine		−0.710	(−2.149–0.730)		
Potassium		0.023	(−0.001–0.048)		
Sodium		−0.022	(−0.044–0.000)		
Urea		0.000	(−0.001–0.000)		
ALT		−0.005	(−0.011–0.002)		
AST		−0.001	(−0.006–0.003)		
Bilirubin		0.662	(0.069–1.254) *	0.138	(−0.752–1.029)
Direct bilirubin		−0.006	(−0.035–0.022)		
Last HBA1C		0.692	(−3.749–5.134)		
O_2_ saturation					
	=>94	R	R		
	90–94	0.943	(−0.841–1.321)		
	<90	1.471	(0.705–2.236) *	0.215	(−1.037–1.466)
Steroids	Yes	1.837	(−0.335–4.010)		
Tocilizumab	Yes	2.815	(0.553–5.078) *	2.689	(−0.757–6.134)
Remedisvir	Yes	1.564	0.043–3.085) *	0.054	(−2.380–2.488)

* *p*-value < 0.05, R: Reference group, COR: Crude odds ratio, AOR: Adjusted odds ratio, CB: Crude B coefficient, AB: Adjusted B coefficient.

## Data Availability

The data that support the findings of this study are available from the first or/and the corresponding author upon reasonable request.
